# What is New in Contact Allergy To Cosmetics for Physicians, Cosmetologists, and Cosmetic Users?

**DOI:** 10.1007/s11882-025-01226-5

**Published:** 2025-10-24

**Authors:** Thanisorn Sukakul, Cecilia Svedman

**Affiliations:** Department of Occupational and Environmental Dermatology, Faculty of Medicine, Lund University, Skåne University Hospital, Jan Waldenströmsgatan 16, pl. 5, Malmö, 20502 Sweden

**Keywords:** Cosmetics, Allergic contact dermatitis, Patch test, Repeated open application test

## Abstract

**Purpose of Review:**

This paper aims to evaluate the burden of contact allergy caused by cosmetic products, identifying the key allergens involved and examining recent regulatory and diagnostic developments. The review addresses which substances commonly induce allergic contact dermatitis and how current trends and emerging allergens impact clinical practice.

**Recent Findings:**

Fragrances remain the most prevalent cosmetic allergens, with numerous compounds capable of triggering sensitization. Recent regulatory improvements in fragrance labeling are helping to reduce exposure to major allergens. Preservatives such as formaldehyde and isothiazolinones have historically caused widespread allergic reactions, but restrictions have lowered their incidence. Hair cosmetic allergens, especially para-phenylenediamine (PPD) and related chemicals, continue to cause significant allergic responses in consumers and professionals. Newly recognized allergens appear in sunscreens, skin lightening agents, and natural ingredients like propolis and carvone. The primary diagnostic methods include patch testing with baseline and specialized cosmetic allergen panels, photopatch testing for photoallergens, and repeated open application tests to support clinical evaluation.

**Summary:**

Contact allergy due to cosmetics is a growing dermatological issue, primarily driven by fragrances and hair dye allergens, with emerging allergens contributing to the evolving landscape. Continued vigilance in diagnosis, improved regulatory measures, and increased reporting are essential to reduce allergy rates and enhance patient care. This review underlines the need for ongoing research and prevention strategies targeting novel cosmetic allergens.

## Introduction

### Cosmetics and Contact Allergy

Cosmetics are, by definition, products applied to the external parts of the body: the skin, hair, nails, and lips [1]. Oral care products are sometimes categorized as cosmetics. Cosmetics are products regulated in many countries, for example, within the European Union [1, 2]. The use of cosmetics has increased and is today common among both females and males, and also in children. Cosmetic products are a frequent cause of contact dermatitis, both irritant and allergic. Many commonly used ingredients can trigger skin allergy in susceptible individuals. As the cosmetic market continues to grow, new ingredients are being introduced, and an increasing number of these have been linked to contact allergy. In addition, consumers often use several products at the same time and in different ways, which can increase the risk of skin reactions, especially when the products are combined or used incorrectly.

Clinically, an individual with contact dermatitis mainly presents with eczema or spongiotic dermatitis, which can vary from subtle dermatitis with symptoms of tingling, itchiness, and redness to more prominent eczematous lesions such as papules, scaly patches and plaques, blisters, lichenification, fissures, and ulcers, which sometimes can be painful. The clinical presentations cannot be used to distinguish between allergic contact dermatitis, irritant contact dermatitis, and other causes of dermatitis, such as endogenous eczema. Clinical dermatitis might be simultaneously caused by both allergic and irritant contact dermatitis, especially from products directly exposed to the skin as topical medications and cosmetic products [3].

Contact allergy to cosmetics is a delayed-type hypersensitivity reaction (type-IV allergy) [4]. To diagnose contact allergy to cosmetics, patch testing with both the baseline series and furthermore with cosmetic-related substances is the gold standard [5]. As cosmetics are often complicated mixtures of ingredients and many substances might be found in several products and even in products that are not cosmetics, a contact allergy per se does not by necessarily explain the symptoms the patient experiences. Thus, to diagnose allergic contact dermatitis, there must exist a positive allergic reaction to the contact allergen, an exposure to the allergen in a sufficient dose to elicit a reaction, a clinical dermatitis appearing on the skin area that has been exposed to the substance, and an appropriate time relationship.

As cosmetics are applied in different ways, the pattern of exposure may vary, and in some cases the source is not as easily identified as in dermatitis caused by leave‑on products, there exists connubial dermatitis when the cosmetic user is usually an individual with close connection to the individual with allergic contact dermatitis, airborne dermatitis and photoallergic contact allergy giving rise to a photoallergic contact dermatitis, a form of delayed-type (type IV) hypersensitivity caused when a substance applied to the skin becomes allergenic after activation by ultraviolet (UV) light. The latter is particularly common in chemicals used in sunscreen cosmetics.

Immediate hypersensitivity reaction (type-I allergy) to cosmetics has also been reported, even though it is not as prevalent as delayed-type hypersensitivity. The most common clinical presentation for type-1 contact allergy to cosmetics is contact urticaria, whereas angioedema and anaphylaxis caused by cosmetics are rare. Non-immunologic contact urticaria is sometimes diagnosed in connection with specific substances used in cosmetics. The delayed-type (type IV) hypersensitivity reaction (allergic contact dermatitis) may sometimes present with clinical features that resemble other conditions. For example, it can mimic type I hypersensitivity reactions seen with hair dyes or lichenoid eruptions associated with oral allergens such as carvone.

Since cosmetics are used from head to toe, dermatitis may appear on any part of the body. However, cosmetic contact allergy is mostly reported and suspected in patients with facial dermatitis. About 30% of patients with facial dermatitis have been reported to have cosmetic-induced facial dermatitis [6]. Patch testing with cosmetic-related allergens is mainly performed in patients with “red face” or facial dermatitis. Females are more likely to have contact allergy to cosmetics than males [6–9]. Contact allergy to cosmetics is also more common among middle-aged patients who are usually exposed to the allergens in cosmetics. However, since cosmetic products have also become popular among children [10, 11], contact allergy to cosmetics is also becoming common in the younger patient group [12–15]. Even occupational allergic contact dermatitis has also been reported due to cosmetic-related contact allergens, such as hand eczema in sellers of cosmetics and beauticians [16, 17].

### Contact Allergy Investigations: How to Diagnose

#### Patch Test: the Gold Standard Investigation To Diagnose Contact Allergy

In order to diagnose a contact allergy, it is important to patch test following the existing guidelines. The test preparations are tested on the skin for 48 h, and the reactions are read thereafter, usually on days 3/4 and 7 [5, 18]. Patch test reading day 7 is particularly important for certain allergens such as acrylates. Many substances used in cosmetics are commercially available as patch test preparations. Common cosmetic contact allergens, especially fragrances and preservatives, are usually included in most of the baseline series; however, the baseline series will not suffice. In case of a suspicion of contact allergy to cosmetics, testing with cosmetic series, suspected products, and their individual ingredients should be performed [19]. The latter is performed as contact allergy to emerging or new substances in cosmetics might be missed since the substances are commercially available with a delay. Even if patch testing is performed with own material, contact allergy might be missed as the dose to elicit a reaction when occluded for 48 h might be insufficient when tested as is or with dilutions.

#### Photopatch Test: A Gold Standard Investigation To Diagnose Photocontact Allergy

When a photoallergic reaction is suspected, the patient must not only be patch tested, but a photopatch test must also be performed. Also, for photopatch testing, there exist guidelines [20, 21]. Two panels of the test preparations are patched on the skin test areas: the irradiated panel, which is irradiated with UVA light, and the non-irradiated panel [20]. The test reactions are compared between the irradiated and non-irradiated sides to provide the diagnosis. Common cosmetic allergens in photopatch test series are ultraviolet filters in sunscreens, this being possible allergens as the UV radiation, when absorbed by the substances, might be more allergenic [22, 23].

#### Repeated Open Application Test: A Useful Test for all

**Fig. 1:**
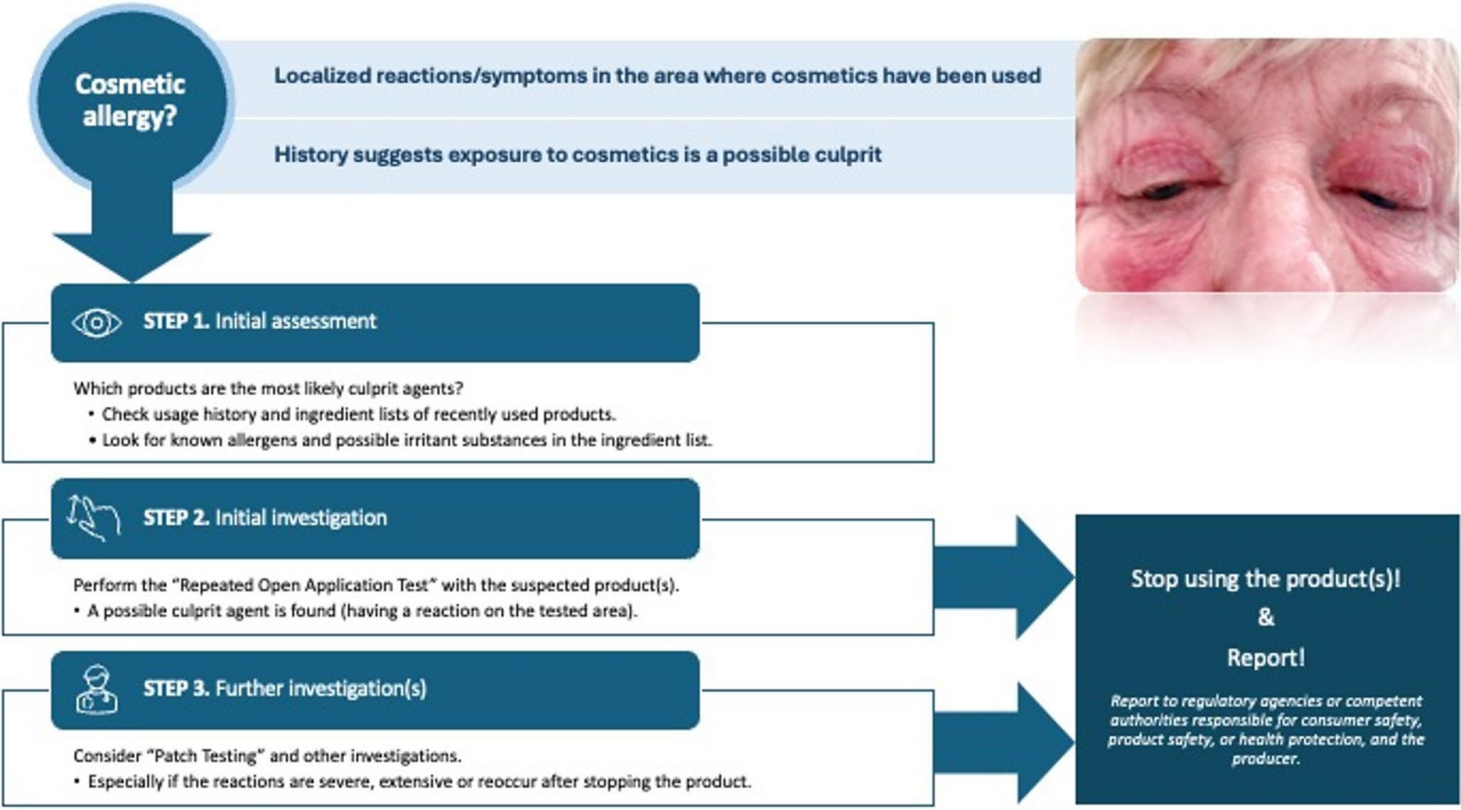
Approach to suspected contact allergy from cosmetic products

The repeated open application test is a clinically useful tool to aid an individual with a possible cosmetic dermatitis [24]. It can be used to provide support for a possible allergic contact dermatitis. The procedure is, however, not standardized. The test aims to evaluate whether the use of cosmetics can elicit a skin reaction on the tested area. Therefore, the procedure should represent the use of cosmetics in real-life situations. Repeated open application test is commonly recommended and mostly useful for leave-on cosmetics. The suspected leave-on product is repetitively applied on an area of the skin, usually on the forearms or around the cubital fossa, about the size of a large coin, for 3–4 weeks. The frequency of application can vary from once a day to a few times a day, mimicking real life. It may also be useful when contact allergy to metal parts of cosmetic product containers is suspected [25]. A rash on the tested area suggests a skin reaction, which could be either an allergic or irritant reaction. Repeated application test can be performed alone without patch testing for product avoidance, but it will then not prove whether the reaction is due to contact allergy. A repeat open application test may also give support that a product, as such, is not the culprit agent, but perhaps the way the product is used [25] or even, when used in research, support safe concentrations for possible allergens [26]. Figure 1 demonstrates an approach to suspected contact allergy from cosmetic products.

## Contact Allergens in Cosmetics

Cosmetic products can be categorized in different aspects by function, by area of application, by product form and texture, or by ingredient type. In almost all kinds of cosmetics, whether they are leave-on or rinse-off products, fragrances and preservatives are often used as mutual ingredients. The trends of fragrance and preservative substances that cause contact allergy have changed over time. Consumers can also be sensitized by other common ingredients in cosmetics, such as emulsifiers and surfactants. Furthermore, other new and prevalent cosmetic ingredients have been in the focus of cosmetic contact allergy. Table 1 summarizes the keys of contact allergy to cosmetics categorized by types of cosmetic ingredients.

**Table 1:** What is new in contact allergy to cosmetics?

History and clinical presentations	
	- Contact allergy to cosmetics is also common in consumers at a younger age. - Dermatitis caused by cosmetics is not limited only to the face, but also on other locations due to the widespread use of cosmetics. - Allergic contact dermatitis to cosmetics may not present only as an eczematous reaction, but also as a suspected type-I reaction or an oral lichenoid reaction - Occupational cosmetic contact allergy (occupational hand eczema) has been reported.
Contact allergens	
Fragrances	- Fragrances and essential oils remain the main contact allergens in cosmetics. - Hydroperoxides of linalool and limonene contact allergies are common; however, the diagnosis of both contact allergy and relevant contact allergy is complicated. - Cosmetic regulation focusing on fragrance use has been updated and will come into force in 2026 and 2028.
Preservatives	- Prevalence of contact allergy to preservatives used in cosmetics has declined due to restricted use of parabens, formaldehyde, and methylchloroisothiazolinone and methylisothiazolinone. - Other preservatives, such as phenoxyethanol, ethylhexylglycerin, and benzyl alcohol in cosmetics, are generally safer.
Skin lightening agents	- Contact allergy to skin lightening agents may be an emerging problem. Repeated open application tests with the suspected products can be useful.
Sunscreens	- Many sunscreen ingredients (UV filters) are allergens and can be photocontact allergens. - New organic ultraviolet filters are not commonly reported as causes of contact allergy.
Acrylates	- An increasing trend of contact allergy to acrylates has been observed. - (Meth)Acrylates (i.e., hydroxyethyl methacrylate) are the major cause of contact allergy due to nail cosmetics. - Sources of exposure to acrylates can be varied, from dental materials, glues, and adhesives. - Regulation on the restricted use of acrylates is needed.
Hair cosmetics	- *para*-Phenylenediamine (PPD) has remained the most common culprit allergen in hair dyes. Other hair dye agents, such as toluene-2,5-diamine or 2-methoxymethyl-p-phenylenediamine, could be alternatives for individuals with PPD allergy. However, cross-reactions and sensitization to the substances are common.
Natural cosmetic ingredients	- Carvone (a fragrance material), propolis (an extract from beeswax), and carmine (a red pigment) are allergenic substances related to the use of lip and oral care cosmetics.
Other excipients	- Surfactants, emulsifiers, and antioxidants (cocamidopropyl betaine, sorbitan sesquioleate/oleate, sodium metabisulfite, glucosides) can cause contact allergy and are still commonly used in cosmetics. - Contact allergy to metals and pigments in cosmetics has been uncommonly reported.
Cosmetic product packaging	- Metal parts of the cosmetic product packaging may contain contact allergens, such as nickel. - Formaldehyde has been reported as a contamination in the cream from the product’s packaging.

### Fragrances

More than 150 fragrance materials are known to be allergenic, causing contact allergy, photo contact allergy, and contact urticaria [27, 28]. Many fragrances are non- or low-allergenic chemicals that can be transformed into other chemical substances, which can become more allergenic and cause contact allergy. Recently, there has been a focus on substances that are categorized as haptens, prehaptens, and prohaptens [29]. A hapten is a sensitizing chemical that can penetrate the skin and bind directly to the protein, resulting in contact allergy. Linalool and limonene are fragrance terpenes that are prehaptens, where the substances themselves are non- or low-allergenic [30]. Nevertheless, after a chemical transformation of the substances by air-oxidation, their oxidation products, “hydroperoxides”, are much more allergenic [30]. Some fragrance materials can act as a hapten and/or a prehapten and/or a prohapten [31].

- Hydroperoxides of linalool and limonene as “prehaptens”: Are we exposed to the allergens?

The most problematic issue in fragrance contact allergy is when the fragrance materials are prehaptens. It is difficult to know whether the prehaptens have been transformed in the cosmetic products to be haptens. The common oxidized terpenes, linalool hydroperoxides and limonene hydroperoxides, are allergenic substances [30]. Contact allergies to linalool hydroperoxides and limonene hydroperoxides have been reported to be very common and could be higher than other fragrances in the baseline series [32]. Linalool and limonene (non-oxidized) are the most common fragrances used in cosmetics and consumer products [33]. However, the degree of exposure to the real haptens, the hydroperoxides, is insufficiently established. The amount of linalool hydroperoxides and limonene hydroperoxides reported in cosmetics can rarely elicit a skin reaction [26]. Therefore, as the whole chain of evidence is lacking, the contact allergies are often deemed with unknown relevance. This is the explanation for why many baseline series do not contain the hydroperoxides for screening purposes, even if the contact allergy is frequently identified. Further studies are still needed to confirm the relation between contact allergy and allergic contact dermatitis. In case of a suspicion of allergic contact dermatitis to fragrances, including the hydroperoxides, performing a repeated open application test can be beneficial.

- Contact allergy to multiple fragrances: A signal for fragrance-free cosmetic use.

Concomitant contact allergies to fragrances are common, and several fragrance markers exist within the baseline series. Patch testing with fragrance mixes I and II, *Myroxylon Pereirae* resin (Peru balsam), and colophonium (a fragrance marker) in most of the baseline patch testing series is a useful and relevant test for patients with contact allergy to fragrances [34]. Patients with contact allergy to more than one individual fragrance material or having a strong reaction to the individual fragrances should be detected by patch testing with the mixes in the baseline series [34]. Patients with multiple fragrance contact allergies are recommended to use fragrance-free cosmetics.

- Updated European Union cosmetic regulation on fragrances: For safer cosmetics.

International Nomenclature of Cosmetic Ingredients (INCI) names of 26 fragrances must be listed on the cosmetics as ingredients when the individual amount exceeds the limits for over a decade to prevent skin sensitization and elicitation [35, 36]. Of these 26 fragrance materials, butylphenyl methylpropional (lilial) and hydroxyisohexyl 3-cyclohexene carboxaldehyde (lyral) have been banned due to their high skin sensitization potency.

In the near future, cosmetics that will enter the European Union market (after 31 July 2026) and those that are already on the European Union market (after 31 July 2028) will be affected by an updated regulation concerning fragrance materials [36]. The European Commission has added 56 new entries of fragrance substances to the Annex, meaning that in total, 80 fragrance materials must be declared [36]. “Parfum” or “aroma” must still be disclosed if the content is lower than the stated amount in the regulation. Furthermore, to prevent a risk of sensitization to fragrances, quantitative risk assessments have been performed to limit the use of known allergenic fragrance materials in consumer products [37, 38]. The trend of contact allergy to fragrances is expected to decline.

### Preservatives

The trend of contact allergy to preservatives in cosmetics seems to have decreased over recent years due to the restriction of the use and changes in cosmetic production. Among previously common preservative allergens in cosmetics, such as parabens, isothiazolinones, formaldehyde, and formaldehyde releasers, these are not as commonly used in cosmetics as in the past.

- Parabens.

The use of parabens in cosmetics has been restricted and banned in the European Union [39, 40]. However, the main reasons for the concerns have not been based only on the skin sensitization risk. Instead, there have been concerns about long-term safety, health risks, and environmental impact, which potentially affect consumer demand for safety products [39, 40]. Parabens might not be restricted or banned worldwide, but the term “paraben-free” has become a positive marketing term, and this may have decreased the use of the preservative elsewhere. Therefore, the prevalence of paraben contact allergy has been reported to be low, which might be due to less skin exposure when compared to the past [7, 41–43]. Consequently, paraben was announced as the contact “non-allergen” of the year in 2019 to avoid the misunderstanding [43].

- Isothiazolinones.

Isothiazolinones, mainly methylchloroisothiazolinone and methylisothiazolinone, are preservatives that have been extensively used in cosmetics. In the 2010 s, there was a pandemic of isothiazolinone contact allergy [44]. Restrictions on methylchloroisothiazolinone and methylisothiazolinone used in cosmetics have had a positive impact on contact allergy since both have been banned in leave-on products [1, 45–47]. The prevalence of methylchloroisothiazolinone and methylisothiazolinone contact allergy has decreased in many countries worldwide after the regulation came into force [48–52]. Even though the use of methylchloroisothiazolinone and methylisothiazolinone in cosmetics is regulated mainly in Europe, they still exist in rinse-off cosmetics produced and sold in the European Union and are available in both leave-on and rinse-off cosmetics in many countries, including the United States, Canada, and Australia. However, the use of methylchloroisothiazolinone and methylisothiazolinone has become less prevalent in cosmetics due to market pressure. However, patch testing with isothiazolinones is still useful in patients with suspected cosmetic contact allergy. Other concerns regarding isothiazolinone contact allergy are that cross-sensitization to other isothiazolinones is common, and their presence in non-cosmetic products [48].

- Formaldehydes and formaldehyde releasers.

Contact allergy to formaldehyde is common, and it has almost always been included in the baseline patch test series worldwide [53–55]. Formaldehyde has been banned, restricted, and discouraged from use in cosmetics worldwide following the European Union Cosmetic Regulation [1]. However, formaldehyde-releasing preservatives are usually allowed in cosmetics with strict limits, except quaternium-15, which has been banned in the countries of the European Union [1]. Consequently, the contact allergy prevalence of formaldehyde and its releasers has decreased [56]. However, cosmetic products may contain a low amount of formaldehyde and cause contact allergy even though it is not declared on the product labelling [57, 58]. Other alternative preservatives that have become commonly used in cosmetics, such as phenoxyethanol, ethylhexylglycerin, and benzyl alcohol, are not as allergenic as the aforementioned substances [7, 48, 59].

### Skin Lightening Agents

Unwanted hyperpigmentation of the skin and the possibility of lightening of areas of uneven pigmentation have become common concerns among consumers and patients with hyperpigmented skin lesions. Hexyl resorcinol, a resorcinol derivative presented in skin-lightening products, can cause contact allergy [60]. Another emerging substance, topical isobutylamido thiazolyl resorcinol (Thiamidol), is an effective anti-pigment ingredient that can serve as an alternative treatment option for melasma [61]. After a few years of marketing, to this date, three cases of allergic contact dermatitis have been reported [62, 63]. Not all skin lightening agents are commercially available for patch testing. Even if they are available, they are not included in broader screening test series nor adequately investigated for the optimized test dose. Thus, this patient group might be underreported [61, 63]. As the use of these products has increased, it is important for the clinician to consider possible adverse skin reactions.

### Sunscreens

Sunscreens have been widely used in all ages to prevent photodamage. Several sunscreen ingredients, which have been in use in cosmetics for a long time, such as octocrylene, benzophenones, p-aminobenzoic acid, derivatives of salicylate, cinnamates, and oxybenzone, have been known to cause contact allergy and photocontact allergy [64].

Newer UV filters have also been reported to be the culprits. Tinosorb S (bis-ethylhexyloxyphenol methoxyphenyl triazine) and Tinosorb M (methylene bis-benzotriazolyl tetramethylbutylphenol), emerging broad-spectrum UV filters, have been reported to cause contact allergy and photocontact allergy [22, 65]. However, the prevalence of contact allergy rates has not been well-established since they have become more in used during recent years. Contact allergy to oleoyl tyrosine, an ingredient in tan-enhancing sunscreens, has also been reported [66]. Ecamsule (Mexoryl SX) and Drometrizole Trisiloxane (Mexoryl XL) contact allergy and photocontact allergy are considerably rare [64, 67]. Physical (inorganic, mineral) UV filters, such as titanium dioxide and zinc oxide, should not cause contact allergy or photocontact allergy due to the larger particle sizes. However, sunscreen products usually contain both physical and chemical (organic) UV filters, which the latter can act as contact allergens [68].

### Acrylates

Acrylates have been frequently implicated in cosmetic allergic contact dermatitis due to their extensive use in nail salons and home-use gel and nail kits. Hydroxyethyl methacrylate, a common acrylate substance in many baseline series, is a well-established allergen in nail cosmetics [69]. The prevalence of contact allergy to methacrylate in nail cosmetics has increased [70]. Other acrylates and isocyanates, such as hydroxyethyl acrylate, ethyl acrylate, and isophorone diisocyanate in nail cosmetics, can also cause contact allergy [71]. Nail glue gel can also contain isobornyl acrylate, which is a common cause of allergic contact dermatitis in diabetic medical devices [72].

The sources of skin exposure to acrylates can also be from dental materials and adhesives, where sensitization and elicitation may occur when the materials are used in the oral cavity or the adhesive part of the devices is attached, and can relate to occupational contact dermatitis (hand eczema) [73, 74]. Patients with contact allergy to acrylates in nail products might not have periungual dermatitis or rash around the nails. They can present with eyelid or facial eczema where they are touched by fingers and nails [71, 75].

### Hair Cosmetics

Hair dyes and hair bleaching agents remain the main causes of contact allergy on the scalp and head and neck area. Para-phenylenediamine (PPD) has remained the most common allergen in hair dye products [76]. Other hair dye substances, such as toluene-2,5-diamine and 2-methoxymethyl-p-phenylenediamine, have become alternatives for patients with PPD contact allergy. However, cross-reactions between different hair dye agents may occur. Even if declared PPD-free, the products might not always be safe for PPD-sensitized individuals [77–79].

### Natural Cosmetic Ingredients

Consumers are at risk of being sensitized and, furthermore, having allergic contact dermatitis, i.e. an elicitation reaction, by natural cosmetic products, as natural ingredients may also be allergenic. Most of the fragrances and essential oils that are common ingredients in products that claim to be natural are common fragrance sensitizers [80, 81]. Other well-established contact sensitizers that are natural extracts are propolis, lanolin, and colophonium. Product labelling is insufficient in products from natural sources, and the product, by being labelled as natural, may be misleading for the consumer. For example, PPD was detected in hair dye products labelled “natural” [80, 82]. Selected examples of natural cosmetic ingredients that may give rise to contact allergy are carvone, propolis, and carmine.

Carvone is a naturally organic compound classified as a terpene used as a fragrance in personal care products. (R)-Carvone is a chemical form of carvone that gives a smell of spearmint and is widely used in oral hygiene products such as toothpaste and mouthwash [83]. The prevalence of contact allergy to carvone has been reported in 0.8% of the consecutive patients tested in the US and Sweden, with an increasing trend [83, 84]. Contact allergy to carvone is highly related to lip eczema (cheilitis) and intraoral lesions [84]. Using toothpaste containing carvone has been reported to have clinical relevance in carvone-sensitized individuals [85]. Carvone has also been reported to be a relevant culprit contact allergen in oral snuff nicotine pouches, causing oral lichenoid lesions [86].

Propolis is a natural resinous substance made by bees, used in cosmetics. Contact allergy rate to propolis has increased, and concomitant contact allergies to fragrances and colophonium are common [87, 88]. However, propolis, being a natural product, is complicated to patch test as it might contain different ingredients when purchased from different geographical regions. At the same time, the products containing the substance can also be produced in different parts of the world. Thus, patch testing with the patient’s own product should be performed. In case the suspicion is high and the patch test result is negative, a repeated open application test should be performed. Most of the cases with contact allergy to propolis, where the allergy has been deemed relevant, have had cheilitis due to exposure to lip cosmetics [89].

Carmine is a red pigment used in cosmetics (CI 75470), which is made from the crushed bodies of cochineal insects [90]. In a study in the US, 3.1% of 4240 patch tested patients had a positive reaction to carmine (2.5% in petrolatum) [90]. Type-1 allergy, including contact urticaria to carmine, has also been reported [91]. This group of patients usually has type-1 food allergy to cochineal and can react to carmine in cosmetics [91, 92]. The relevant sources of carmine contact allergy are makeup, mainly lipsticks, since they may contain higher amounts of the red pigment of carmine [90, 91].

### Other Excipients: Emulsifiers, Surfactants, Antioxidants, Metals, and Pigments

Other common ingredients in cosmetics that have been reported to cause contact allergy are emulsifiers and surfactants. Among emulsifiers used in cosmetics, contact allergy to cocamidopropyl betaine and sorbitan sesquioleate/oleate has sometimes been reported [15, 93–95]. Glucosides (i.e., decyl glucoside, lauryl glucoside, coco glucoside) serve as both surfactants and emulsifiers and can cause contact allergy, and cross reactions between glycosides are expected [96–98]. Sodium metabisulfite is a chemical compound in the sulfite group (sulfites) commonly used in cosmetics and personal care products, primarily as a preservative and antioxidant [99]. Sulfites have been nominated to be the allergen of the year 2024 [99]. However, there are still doubts about the test reactions and clinical relevance in patients with contact allergy to sodium metabisulfite [100]. Metals and pigments are uncommonly reported to be causes of contact allergy to cosmetics [101, 102].

### Allergens in Cosmetic Product Packaging

Cosmetic product packaging can also be a source of contact allergen exposure. Many beauty metal tools were found to release nickel and cobalt in levels that can cause allergic contact dermatitis [103]. Metal tips of eye cream product containers used to massage the eyelids have also been found to be the cause of allergic contact dermatitis to nickel [25]. Formaldehyde has been reported to be a contamination in cosmetics even though it is not labelled, as it has been found to be released from the containers [104].

## Conclusions

Given the wide variety of cosmetic products used and the ongoing demand for new formulations to meet emerging consumer needs, new ingredients and exposure scenarios continue to emerge. Increased exposure to the new cosmetic ingredients may elevate the risk of sensitization and elicitation among consumers. In case of a suspicion of contact allergy to cosmetics, a repeated open application test is useful and can be performed by the cosmetic users. To identify the exact culprit allergen(s), a patch test must be done. Changes in the use of cosmetic ingredients mean that consumers, cosmetologists, and physicians need to be aware of emerging allergens that might not have been reported or investigated earlier. In many countries, most likely the number of actual adverse skin reactions to cosmetics is under-estimated due to the fact that a reaction is not reported. User-friendly report systems and more important recommendations on adequate patch testing when reports are filed would benefit both the consumer and the producer, enhance useful cosmetic regulations, but more importantly, facilitate research into alternative substances that are low- or non-allergenic

## Key references

- Sukakul T, Bruze M, Svedman C. Fragrance Contact Allergy - A Review Focusing on Patch Testing. Acta dermato-venereologica. 2024;104:adv40332.
  - It highlights that fragrance contact allergy is still common and best diagnosed through patch testing using specific and updated fragrance markers. New EU regulations on fragrances used in cosmetics will soon come into force, which will help to prevent the consumers from fragrance contact allergy caused by cosmetics.
- Isufi D, Jensen MB, Kursawe Larsen C, Alinaghi F, Schwensen JFB, Johansen JD. Allergens Responsible for Contact Allergy in Children From 2010 to 2024: A Systematic Review and Meta-Analysis. Contact dermatitis. 2025;92(5):327 − 43.
  - This study emphasizes that early exposure to cosmetic allergens has increased, partly driven by factors such as social media influence on pre-teens’ cosmetic use, making cosmetics a significant source of allergens in contact allergy in children.
- Sukakul T, Bruze M, Mowitz M, Kiuru A, Svedman C. Allergic Contact Dermatitis to Linalool Hydroperoxides: Pitfalls in the Diagnostic Process-Findings from a Repeated Open Application Test Study. Dermatitis : contact, atopic, occupational, drug. 2024;35(4):373-9.
  - Although the prevalence of contact allergy to linalool hydroperoxides is high, repeated exposure to real-world concentrations of the substances in creams rarely triggers allergic contact dermatitis. It highlights diagnostic challenges, including the variable clinical relevance of positive patch tests and the difficulty in linking exposure from consumer products to allergic reactions.

## Data Availability

No datasets were generated or analysed during the current study.
